# Intravenous Delivery of RNA Encoding Anti-PD-1 Human Monoclonal Antibody for Treating Intestinal Cancer

**DOI:** 10.7150/jca.63991

**Published:** 2022-01-01

**Authors:** Lipei Wu, Weiwei Wang, Jiale Tian, Chunrun Qi, Zhengxin Cai, Wenhui Yan, Shihai Xuan, Anquan Shang

**Affiliations:** 1 Department of Laboratory Medicine, Dongtai People's Hospital & Affiliated Dongtai Hospital of Nantong University, Yancheng 224200, P.R. China.; 2 Department of Laboratory Medicine, Shanghai Tongji Hospital, School of Medcine, Tongji University, Shanghai 200065, P.R. China.; 3 Department of Pathology, Tinghu People's Hospital of Yancheng City, Yancheng 224001, P.R. China.; 4 Department of Laboratory Medicine, Tinghu People's Hospital of Yancheng City, Yancheng 224001, P.R. China.

**Keywords:** mRNA, *In vitro* transcription, Lipid nanoparticle, Anti-PD-1 antibody, Protein replacement therapy

## Abstract

Recently, antibody-based therapeutic agents are becoming most leading biologics for treating many diseases, especially for cancer. However, large-scale application of antibody drugs is still hampered by high cost and complex technical process. Endogenous expression of proteins or antibodies can be achieved by applying *in vitro* transcription (IVT) technique to produce mRNA and then deliver into body, which supplies opportunity to avoid many disadvantages in antibody production as well as clinical applications. Here, we designed the IVT-mRNA encoding the Pembrolizumab, as a commercial anti-PD-1 monoclonal antibody (mAb). The *in vitro* functional properties and *in vivo* antitumor activities of the Pembrolizumab expressed from mRNA were both assessed. Maximized expression level of the Pembrolizumab from IVT-mRNA was achieved via optimizing the usage of signal peptide and molar ratio of heavy/light chain. Then the mRNA was further formulated by lipid nanoparticle (LNP), which enable efficient *in vivo* delivery and protect mRNA from degradation. Intravenously delivered the single dose of mRNA-LNPs in mice resulted in duration of serum Pembrolizumab level over 25 μg/mL more than 35 days. Pharmacokinetic study exhibited significantly enhanced drug exposure of mRNA-encoded mAbs compared with direct injection of Pembrolizumab at same dose. Chronic treatment of the tumor-bearing mice with LNP-encapsulated Pembrolizumab mRNA effectively downregulated the growth of intestinal tumors and improved the animal survival. In brief, our present research demonstrated that the application of LNP-encapsulated IVT-mRNA can change the human body into a protein drug manufacturing site to express full-size mAbs for treating cancer and hold potential to be a novel alternative to protein-based therapies.

## Introduction

Antibody-based drug therapies have huge impact on the treatment of diseases, such as chronic inflammations, autoimmune diseases and tumors, and are also very promising for commercial applications 1, 2. However, the large-scale production protocol and quality supervision of antibodies are too complex, and a brand-new production protocol needs to be designed for each unique sequence of antibodies 3, 4. In addition, the preclinical development cycle of antibody drugs also takes at least 1-2 years 5, 6. In some cases, the mAbs are easy to be rapidly cleared and degraded by the proteases, which necessitates frequent injections of drugs, and bring higher medical fee as well as more side-effects 7-9.

As an alternative approach, researchers have found that antibodies with intact structure and bioactivities can be produced *in vivo* by delivering mRNA prepared by IVT technique 10, 11. This IVT-mRNA-based alternative method can use the human body as a 'manufacture factory' for antibodies production, which can not only get rid of the complex processes, such as mammalian cell culture and purification, in the traditional antibody industry, but also complete the post-translational modification closer to human needs in somatic cells, which will theoretically produce better biological activity 10, 12. Moreover, with the breakthrough of preparation technology for targeted delivery of mRNA, especially for lipid-based nanoparticles (LNPs), the expression of antibodies or other therapeutic proteins in liver tissues or other tumor tissues has been realized 13-15. Rapid and accurate increase of drug concentration at the site, where the antibody exerts its efficacy, can theoretically achieve a lower dose and improve the efficacy compared with the traditional intravenous infusion of antibody 16. Recent researches reported that the application of base modification, such as N1-methyl-pseudouridine, can effectively enhance the stability and reduce immunogenicity of mRNA, respectively 17, 18. In addition, it is very important that IVT-mRNA expression is completed in the cytoplasm without entering the nucleus, which minimizes the risk of inserting into the genome relative to DNA-based therapies 17.

PD-1, as one member of B7 family, is also an immune checkpoint which is mainly expressed on the activated T-cells with ligands of PD-L1 and PD-L2 19, 20. Upon ligation, a negative pathway is promoted to inhibit the function of activated T-cells via down-regulating the signaling of TcR/CD28 21. The expression of PD-L1 on the surface of tumor cells has become a driver for tumor growth caused by tumor escape from immune cell pursuit 22. Thus, the PD-1/PD-L1 pathway is seen as an important mechanistic axis adopted by tumors to facilitate tumor escape 22, 23. Pembrolizumab was one of the first batch of anti-PD-1 therapeutic mAbs to show significant clinical anti-tumor efficacies and also exhibit promising safety profiles, and were approved for the sales on the market by FDA in 2014 24.

In present research, we designed and established an LNP-based IVT-mRNA system for delivery of a full-size anti-PD-1 mAb, Pembrolizumab, and demonstrated that the mRNA-encoded mAbs hold similar *in vitro* bioactivities and functionalities to the Pembrolizumab. We also evaluated the mRNA-LNPs to express full-size Pembrolizumab via liver tissues as a therapeutical strategy for treating cancer, which may also offer an alternative approach for other antibodies for more wide range of clinical applications.

## Materials and methods

### Reagents and animals

AML-12 cells (Lot#: HTX2091) and MC38 cells (Lot#: HYC0116) as a kind of mouse hepatocytes and mouse colon cancer cells, respectively, were both bought from Shanghai Bangjing Industrial Co., Ltd (Shanghai, China). Pembrolizumab was bought from Selleck Chemicals LLC (Texas, USA). Human IL-2, IFN-γ ELISA kits, recombinant PD-1, PD-L1 and PD-L2 were purchased from R&D System (Minnesota, USA). C57BL/6 or NOD/SCID mice, human PD-1 (hPD-1) knock-in (C57BL/6 background) mice were bought from Shanghai Model Organisms Science and Technology Co., Ltd. (Shanghai, China). All the animal studies were approved by the Animal Use and Care Committee.

### Cell culture

AML-12 cells were grown in a 1:1 mixture of DMEM and Ham's F12 with Insulin-Transferrin-Selenium (ITS-G) medium supplement, 100 U/mL penicillin-streptomycin (Pen/Strep), and 10% fetal bovine serum (FBS) (Gibco). All cell lines were cultured at 37°C in 5% CO_2_. When the cells reached 70%-80% monolayer, they were detached from the flask using 0.25% Trypsin-EDTA solution and split 1:5.

### Cloning of template vectors and *in vitro* transcription of mRNA

The mRNAs encoding the heavy chain and light chain of Pembrolizumab were produced via using the T7 RNA polymerase on the linearized plasmid, which was supplied by Genescript (Nanjing, China), with 5' cap (Cap1) and a 3' poly A tail containing more than 110 successive adenines. The 1-methylpseudourine-5′-triphosphate was applied to instead of UTP to obtain modified mRNA. mRNAs were further purified by using MEGAClear RNA purification kits (Life Technologies, USA), and finally stored in the RNase-free water. The purity of prepared mRNA was further analyzed via bioanalyzer (Agilent, USA).

### Lipid-nanoparticle encapsulation of Pembrolizumab mRNA

mRNAs encoding the heavy chain and light chain of Pembrolizumab were collectively encapsulated in LNPs via a self-assembly process. The aqueous solution of mRNA was adjusted to pH = 4.0 and then rapidly mixed with the lipids dissolved in ethanol. The LNPs were prepared via using the phosphatidylcholine, ionizable cationic lipid, PEGylated lipid and cholesterol at a ratio of 10:50:1.5:38.5 (mol/mol). The LNPs encapsulated mRNA was diluted at final concentration of ~0.3 mg/mL and stored at 2-8 °C.

### Affinity and binding specificity to PD-1 molecules

The binding affinities of Pembrolizumab from mRNA or DNA were detected using BIAcore (Cytiva, USA). Antibodies were coated with CM5 series biosensor chip at 5 μg/mL with the serially diluted human PD-1 (2X from 25 nM to 0.39 nM) flowed in the running buffer. The HBS-EP buffer was used as running buffer, containing 0.01 M HEPES, 0.15 M NaCl, 3 mM EDTA, pH 7.4, while the regeneration buffer contained 10 mM glycine-HCl and the pH was 1.5~2.0. Further experimental details were performed according to the user's manual.

### *In vitro* functional validation assays

The CHO cells overexpressed the human PD-1 were engineered and applied to detect the binding affinity of two sources of Pembrolizumab. The CHO overexpressed human PD-1 cells were seeded into the U-bottom plates then treated with Pembrolizumab at final concentration between 0.003 to 50 nM. Those Pembrolizumab binding to CHO overexpressed human PD-1 cells were measured with PE labeled mouse anti-human Fc mAb (PD1-HP2F2, Acro biosystems) and the EC_50_ values were calculated via the GraphPad Prism 8.4.

Further blocking assay of human PD-L1 and PD-L2 to the CHO stably expressed human PD-1 cells was assessed. Different concentrations of two kinds of Pembrolizumab were mixed with human PD-L1 or PD-L2 and then incubated with prefixed CHO human PD-1 cells, the binding of PD-L1 or PD-L2 to CHO overexpressed human PD-1 cells were measured using ELISA method and the IC_50_ values were calculated via the GraphPad Prism 8.4.

### *In vivo* expression validation in mice

The female C57BL/6 or NOD/SCID mice used in pharmacokinetic evaluation were obtained and raised in the animal facility of SLen biotechnology Co., Ltd. (Shanghai, China) with the condition of pathogen-free. LNP-Pembrolizumab mRNA at the doses of 0.2, 0.6 and 2 mg/kg were intravenously (i.v.) treated to the mice via tail veins. The mouse blood was collected from orbit or tail vein and then sits the samples at 4°C for 30 min, and furtherly centrifuged at 1000 g for 15 min. Serum was collected and applied for further ELISA detection or the purification of antibodies.

### MC38 *in vivo* tumor model in human PD-1 knock-in mice

All human PD-1 knock-in female mice were raised in the animal facility of SLen biotechnology Co., Ltd. (Shanghai, China) with the condition of pathogen-free. MC38 cells were diluted into the final concentration of 5*10^6^ cells/mL via PBS and then were s.c. implanted into the right flank of human PD-1 knock-in mice. About 10 days post implantation, the human PD-1 knock-in mice were randomly assigned with mean tumor volume ~ 80 mm^3^. On the day 11 and 23 post-implantation, the hPD-1 knock-in mice were i.v. injected with empty LNPs or mRNA-LNPs at the doses of 0.2, 0.6 and 2.0 mg/kg, using protein antibody at the dose of 10.0 mg/kg as positive control which was injected at day 11, 17, 23 and 29. The body weight and tumor volume were measured every three days, and tumor-bearing mice were euthanized when the body weight loss was over >20%. The tumor size was detected using a standard digital caliper and the tumor growth inhibition (TGI) (%) was calculated as follow: 100% × (Tumor volume of mice treated with empty LNPs - Tumor volume of mice treated with agent)/ (Tumor volume of mice treated with empty LNPs -Tumor volume of mice treated with empty LNPs before dosing).

### Statistical Analysis

One-way ANOVA followed by Tukey's post hoc test was applied for multiple comparisons analysis. Two-way ANOVA followed by the Sidak multiple-comparisons test was applied for repeated measures analysis.

## Results

### Design and Optimization of Pembrolizumab mRNA for LNP Delivery

**Figure 1:**
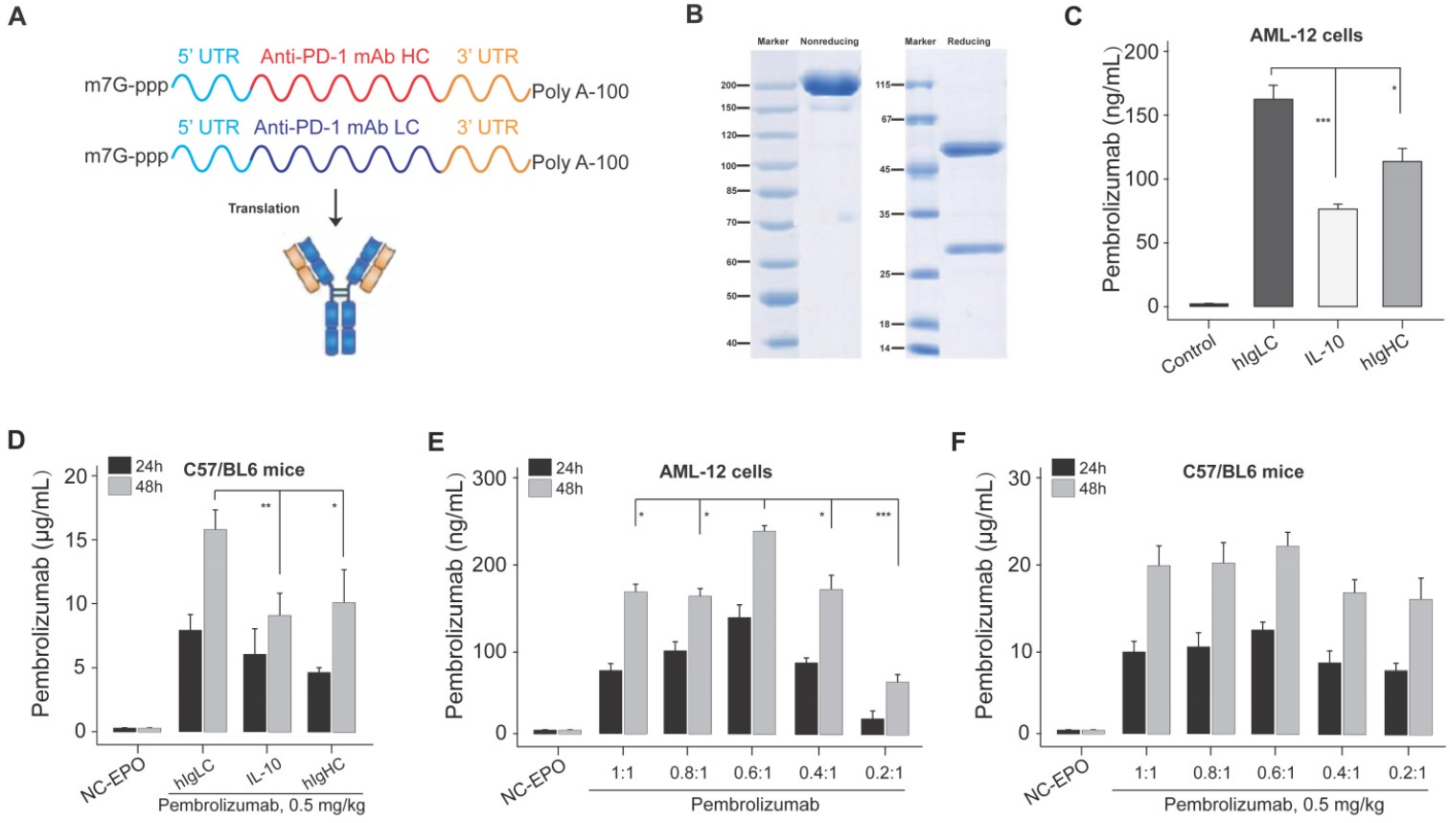
Design and optimization of Pembrolizumab mRNA for LNP delivery. (A) Schematic of the structure of mRNA-encoded the heavy and light chains of the Pembrolizumab. (B) SDS-PAGE detection of the Pembrolizumab translated from mRNA in supernatants from Expi293F cells. (C) The expression levels of Pembrolizumab in supernatants of AML-12 cell transfection with mRNA-encoded Pembrolizumab with three different SPs for 24 h. (D) The serum levels of Pembrolizumab in the C57BL/6 received the single injection of 0.5 mg/kg LNP- Pembrolizumab mRNA with three different SPs. (E) The serum levels of Pembrolizumab in the C57BL/6 received the single injection of 0.5 mg/kg LNP- Pembrolizumab mRNA with different molar ratios. *p < 0.05, **p < 0.01, ***p < 0.001. One-way ANOVA with Tukey's post hoc test. Error bars show the SEM, n=10.

As shown in Figure 1A, the intact mRNA molecule of both heavy and light chain is composed of a 5' cap structure, 5' and 3 ' UTRs, a protein-coding sequence region, and a Poly A tail. In the present study, the cap of our designed mRNA was 2 '-O-methylated Cap0 (Cap1), which can achieve significantly enhanced mRNA translation efficiency and* in vivo* stability. At the same time, the partial sequences of cytomegalovirus immediate early 1 (IE1) and human growth hormone gene were selected as 5 ′ and 3 ′ UTR of mRNA sequences, respectively, and both of which have been reported to promote the efficient expression of proteins *in vivo*. In addition, the length of Poly A tail was approximately 115 nt.

The signal peptide (SP) is a short polypeptide chain containing 5-30 amino acids which directs the translocation of newly synthesized secretory proteins to the secretory pathway. Although there are great differences in SP sequences between different species or proteins, they can often replace each other in the function of guiding the transmembrane transfer of secretory proteins. Based on the above selected mRNA structures, we optimized the SP of light chain and heavy chain sequences in the protein coding sequence region via applying three SPs which originate from human immunoglobulin kappa light chain (hIgLC), interleukin-10 (IL-10) and human IgG1 heavy chain (hIgHC) for the expression validation in AML-12 cell lines* in vitro*. We prepared the linearized DNA template for the IVT of mRNA encoding Pembrolizumab's heavy and light chains with three SPs, respectively, and then DNA sequence was transcribed into six intact mRNA sequences.

Firstly, the integrity of antibody in the supernatant of Expi293F cells transfected with mRNA-encoded the HC and LC of Pembrolizumab was confirmed via the SDS-PAGE (Figure 1B). Then these mRNA with three SPs were paired and transfected into AML-12 cells, and the concentration of intact Pembrolizumab in cell supernatant were measured by ELISA method. As a result, the mRNA containing hIgLC SP resulted in higher expression of Pembrolizumab in all three cell lines compare to other conditions (Figure 1C). Rapid expression test in normal C57BL/6 mice also demonstrated that SP, hIgLC, was more effective for antibody expression (Figure 1D). Therefore, the mRNA with hIgLC SP was selected and applied in the subsequent optimization research.

We further explored the effect of different molar ratios of heavy and light chains (HC/LC) on antibody expression using the same SP. Previous reports indicated that translation rate of protein decreases as the mRNA length increase 25. Thus, we speculated the relatively slower heavy chain expression rates compare to that of light chain which may limit the assembly and secretion of IgG antibody due to the two times longer sequence. Then, we subsequently explored effects of decreased ratio of HC/LC ratio on the enhanced synthesis of IgG antibody. *In vitro* transfection of AML-12 cells with the HC/LC molar ratios of the mRNA encoding the Pembrolizumab in of 1:1, 0.8:1, 0.6:1, 0.4:1 and 0.2:1 was conducted, and the results demonstrated that the HC/LC molar ratio of 0.6:1 resulted in the highest antibody expression compared with others (Figure 1E). Further *in vivo* results did not exhibit a significant higher expression of 0.6;1 compared with others, although that was slightly higher than 0.8:1 and 1:1 (Figure 1F). After comprehensive consideration, the mRNA of with hIgLC SP at the HC/LC molar ratio of 0.6:1 was chosen in the subsequent studies.

### *In vivo* expression and functionality of mRNA-encoded antibodies

**Figure 2:**
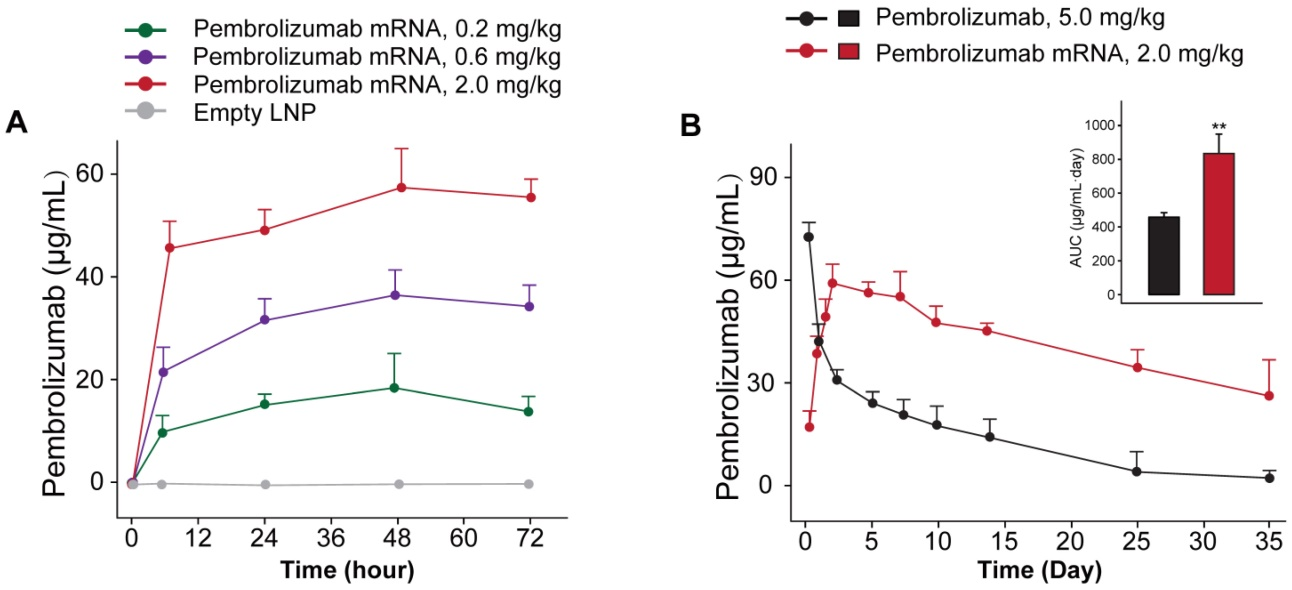
*In vivo* expression of LNP-Pembrolizumab mRNA. (A) Serum concentration of Pembrolizumab in C57BL/6 mice received single injection of LNP-Pembrolizumab mRNA at three doses. (B) Pharmacokinetic test of Pembrolizumab (10 mg/kg) and mRNA-LNPs encoded Pembrolizumab (2mg/kg) in C57BL/6 mice. *p < 0.05, **p < 0.01. All the data were showed as Mean ± S.E.M, n=10.

**Figure 3:**
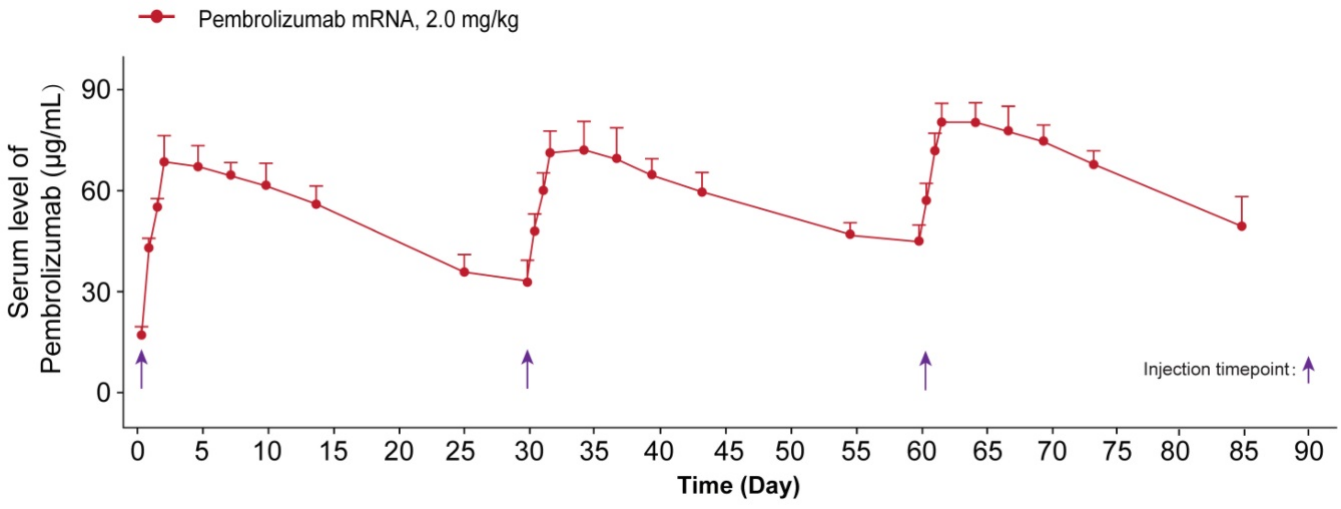
The sustained serum concentration of Pembrolizumab in the NOD/SCID mice received the repeated IV dosing of LNP-mRNA encoded Pembrolizumab. All the data were showed as Mean ± S.E.M, n=10.

We further investigated whether the delivery of optimized Pembrolizumab mRNA is capable of expressing intact antibodies in rodent animals. Those normal C57BL/6 mice received the single injection of formulated Pembrolizumab mRNA at the doses of 0.2, 0.6 and 2 mg/kg were well tolerated without obvious side effects. As showed in Figure 2A, a clearly dose-dependent upregulation of the serum level of Pembrolizumab at 6h, 24h, 48h and 72h after administration were observed. In order to compare the pharmacokinetic parameters of endogenously translated Pembrolizumab with the Pembrolizumab from CHO cell sources, we administered a single injection of 10 mg/kg Pembrolizumab and 2 mg/kg formulated Pembrolizumab mRNA to C57BL/6 mice. As the results showed in Figure 2B, serum concentration of Pembrolizumab antibody peaked at the first timepoint (0.25 h) after injection, and then dramatically decreased within the next 3 days, and continued to slowly decrease over next five weeks. In contrast, serum levels of Pembrolizumab, which were endogenously translated from formulated mRNA, peaked at 48h (~60 μg/mL) and remained over 20 μg/mL at least 35 days after single injection. Furthermore, the delivery of LNP-Pembrolizumab mRNAs also resulted in a higher area under the curve (AUC_0-35days_) than that of direct injection of Pembrolizumab antibody. By administering 2.0 mg/kg of LNP-mRNAs intravenously to immunodeficient mice once a week, an almost consistent Pembrolizumab concentration-time profile can be observed after each injection, indicating that continuous injection of the encoded mRNA in mice is able to stably and continuously express antibodies (Figure 3).

### Functional validation of mRNA-encoded antibodies

To investigate whether the Pembrolizumab produced using optimized mRNA *in vivo* could retain its intact activity of the commercialized Pembrolizumab antibodies from CHO sources, we firstly studied the affinity and binding specificity of the antibodies from two sources, and the blocking potency of PD-1 to PD-L1/L2 as well as the enhancing the T cell functions.

**Figure 4:**
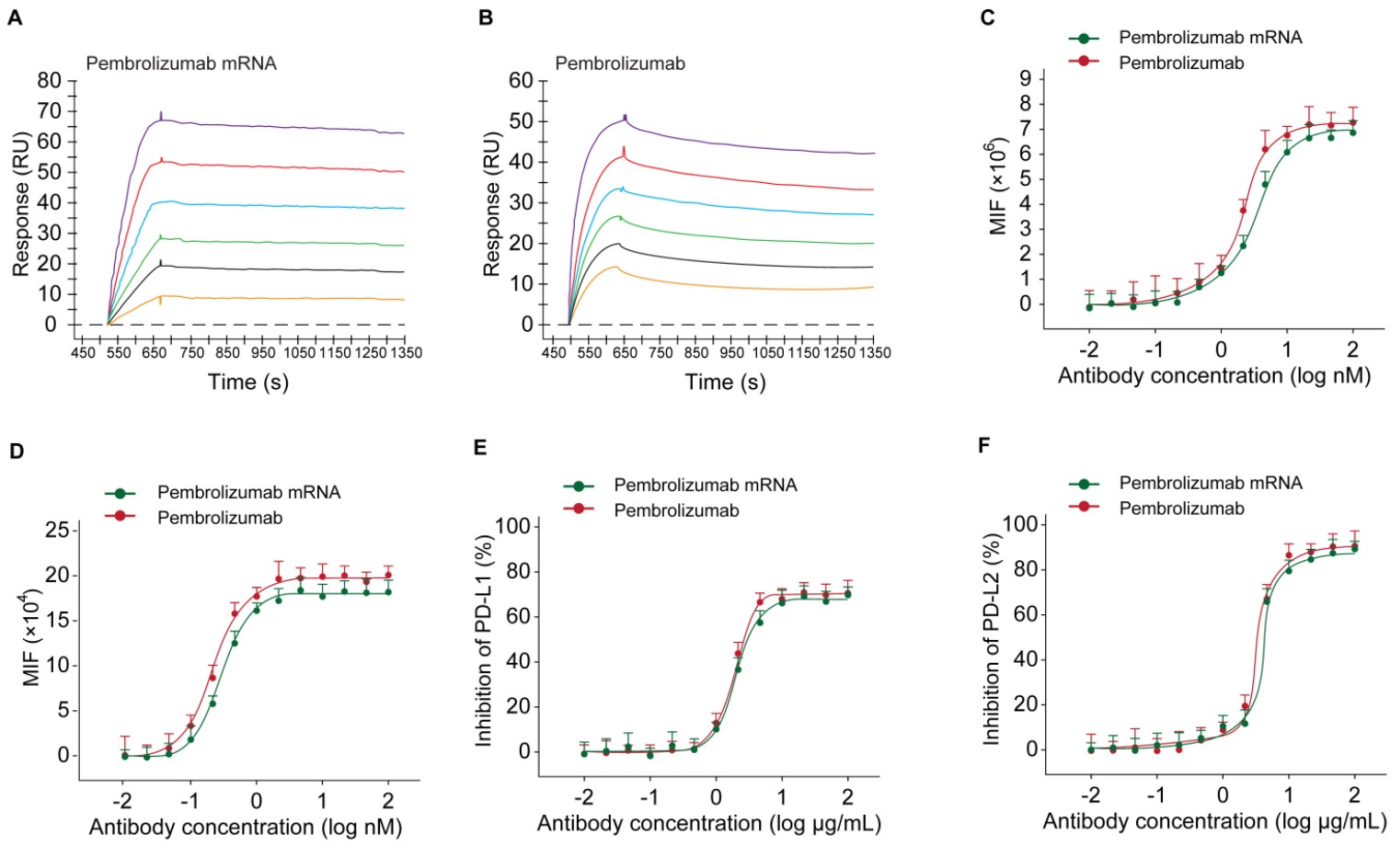
Binding affinity and ligand blocking measurements of Pembrolizumab from mRNA or CHO sources to PD-1 by SPR (Biacore) and cell-based assays. (A-B) Association and dissociation of Pembrolizumab to hPD-1 by SPR. (C) Affinity of Pembrolizumab to CHO-hPD-1 cells. (D) Affinity of Pembrolizumab to activated CD4^+^ T cells. CHO-hPD1 cells-based ELISA with fixed concentrations of human PD-L1 (E) or PD-L2 (F). All the data were showed as Mean ± S.E.M, n=3.

At first, the results of SPR detection exhibited similar human antigen binding affinities of the Pembrolizumab from mRNA or CHO cell sources with the KD of 0.25 nM and 0.61 nM for human PD-1, respectively (Figures 4A-B). Further cell based binding tests on the CHO cell stably expressing hPD-1 were performed with the similar EC_50_ values of Pembrolizumab from mRNA or CHO cell sources (Figure 4C). The binding of Pembrolizumab from different sources and hPD-1 were also demonstrated via the FACS staining with the activated CD4^+^ T-cells, and results also did not show significant difference (Figure 4D, *p*>0.05). PD-L1/L2 blocking tests were performed by application of PD-L1 and PD-L2, as well as antibody treated CHO cell stably expressing hPD-1. As shown in Figure 4D, the Pembrolizumab from both two sources could efficiently inhibit the binding between hPD-1 and PD-L1/L2, with the extremely low IC_50_ at 0.36/0.67 μg/mL for Pembrolizumab and 0.41/0.89 μg/mL for Pembrolizumab mRNA, respectively. Through the above studies, mRNA-derived pembrolizumab held the similar binding and blocking activities to the antibody with same amino acid sequence derived from CHO cells.

**Figure 5:**
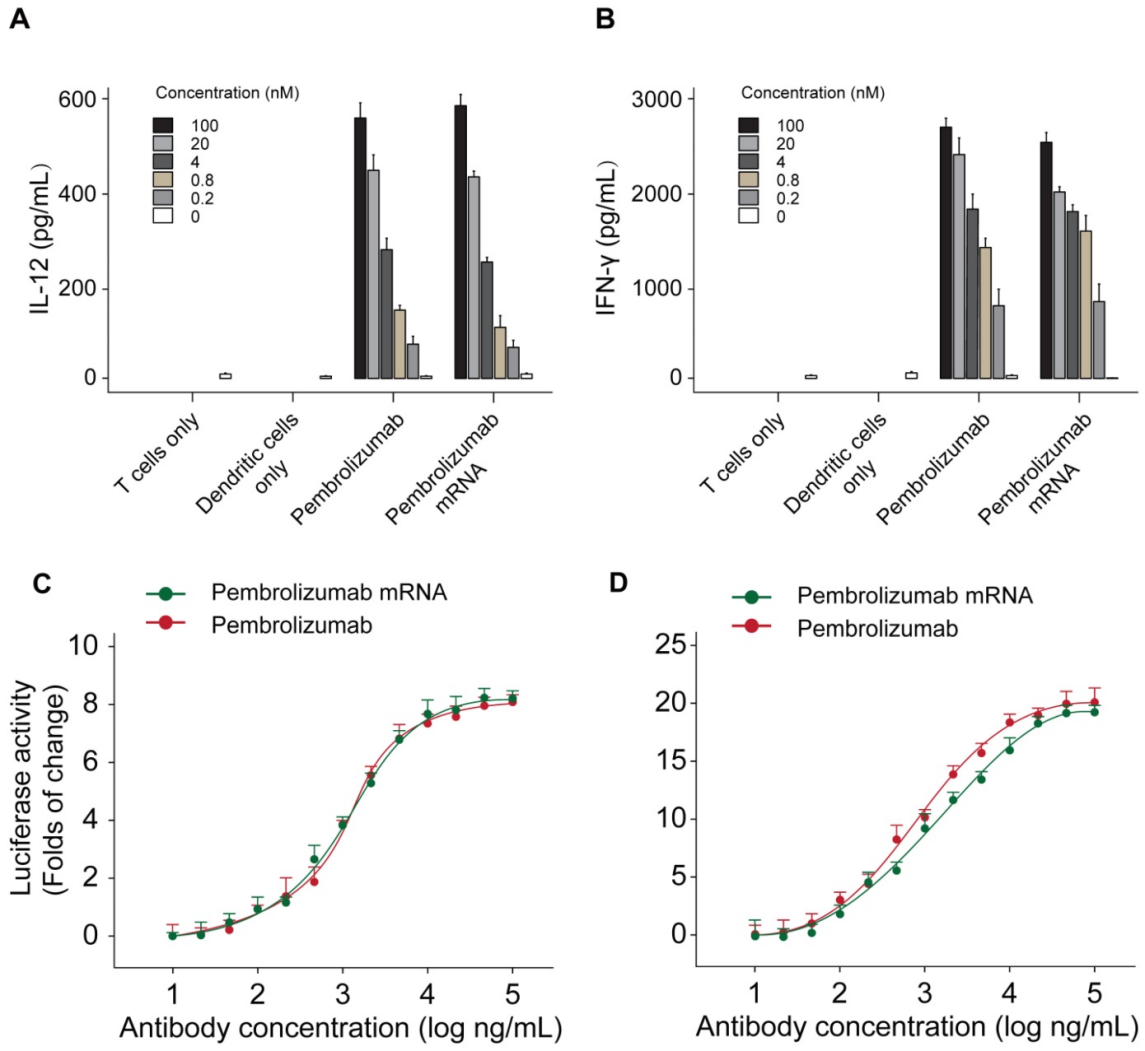
Functional activity of Pembrolizumab from mRNA or CHO sources in cell-based bioassays. Mixed lymphocytes reactions and effects of Pembrolizumab on the activation of T cells. moDC were generated and mixed with CD4^+^ T cells from a different donor for 5 days before detection of IL-2 (A) and IFN-γ (B) secretion by ELISA. (C-D) Luciferase reporter assay. All the data were showed as Mean ± S.E.M, n=3.

The functional activities of Pembrolizumab from two sources were further assessed by applying a mixed lymphocyte reaction (MLR) assay. Monocyte derived dendritic cells were prepared with allogeneic CD4^+^ T-cell and two kinds of Pembrolizumab. As showed in Figures 5A-B, the mRNA translated Pembrolizumab significantly increased the expression levels of IFN-γ and IL-2 in concentration-dependent manners, which were similar to the Pembrolizumab from CHO cell source. In addition, both of two kinds of Pembrolizumab induced comparable potency to activate NFAT (Figure 5C) in a luciferase reporter system which is under control of an NFAT promoter.

### *In vivo* functional validation of the optimized Pembrolizumab mRNA construct

**Figure 6:**
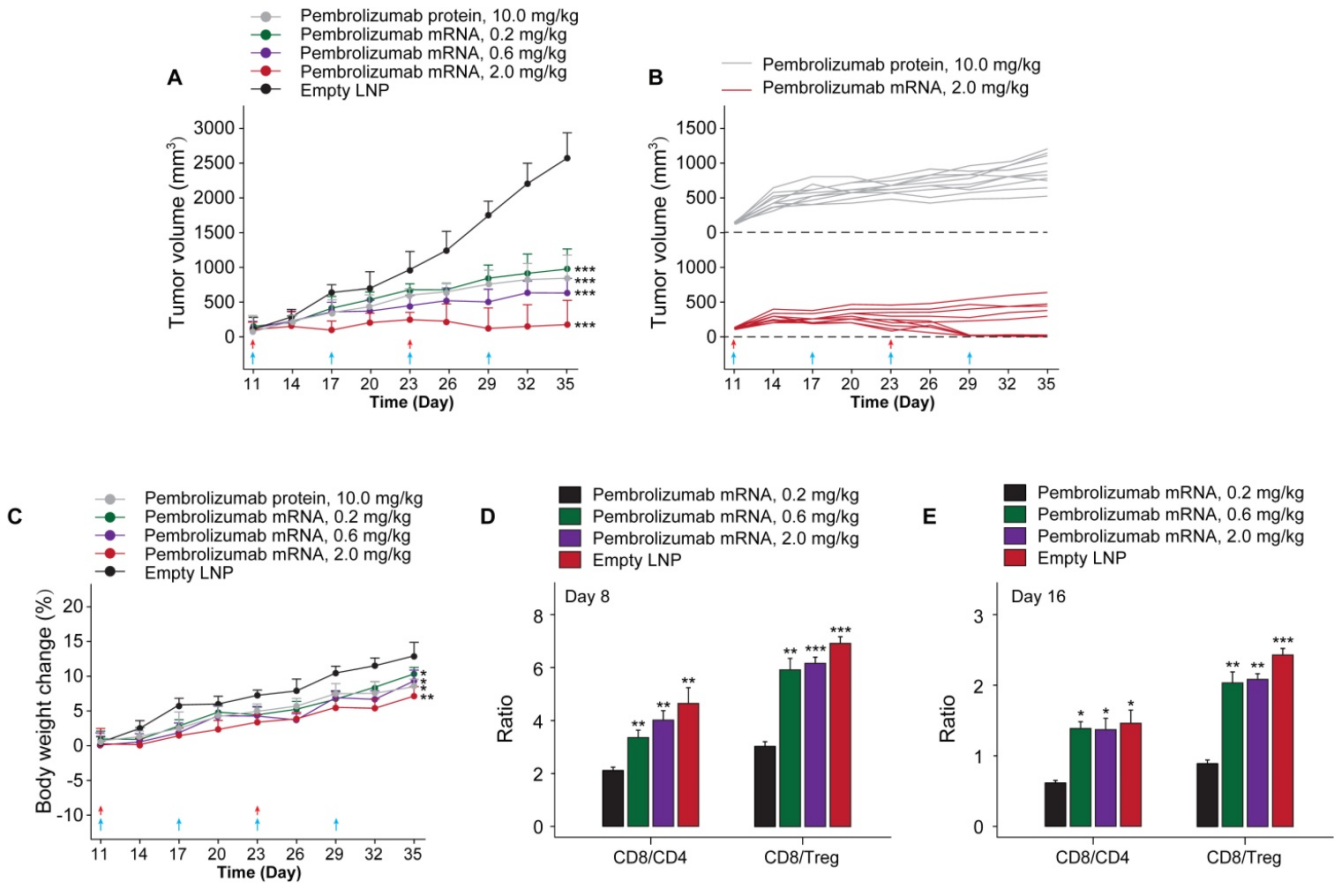
The* in vivo* anti-tumor efficacy test of LNP-Pembrolizumab mRNA in hPD-1 knockin mouse model. (A) Tumor growth inhibition (TGI) of MC38 tumors in hPD-1 knockin mice treated with LNP-Pembrolizumab mRNA at three doses using the Pembrolizumab antibodies and empty LNPs as positive control and negative control, respectively. Red and blue arrows indicated the injection day of LNP-Pembrolizumab mRNA or Pembrolizumab proteins, respectively. (C) Effects on body weight changes. Changes of tumor infiltrating in CD8^+^ T cells/CD4^+^ T cells ratio and CD8^+^ T cells/Treg cells ratio on (D) day 8 and (E) day 16. *p < 0.05, **p < 0.01, ***p < 0.001. All the data were showed as Mean ± S.E.M, n=10.

In order to investigate whether the *in vivo* mRNA-translated Pembrolizumab could retain the anti-cancer activities, we assessed the protective and inhibitory effects on animal survival and tumor growth, respectively, in a therapeutic human PD-1 knock in MC38 tumor bearing mouse which were weekly treated with Pembrolizumab mRNAs at the doses of 0.2, 0.6 and 2.0 mg/kg for 6 weeks, using the Pembrolizumab antibodies at 10 mg/kg as positive control, and tumor growths were recorded. At the beginning of chronic experiment, mean tumor volume in all groups at day 10 was ~100 mm^3^. As the result showed in Figure 6A, empty LNPs treatment exhibited a slight effect on TGI. In addition, we found the significantly enhanced TGI in all mRNA-LNPs treated groups with 0.2 mg/kg for 68%, 0.6 mg/kg for 76% and 2.0 mg/kg for 91.5%, respectively, while Pembrolizumab group was 70.1%, compared to the empty LNPs. Specially, tumor disappeared in 5 of 10 mice treated with 2.0 mg/kg mRNA-LNPs. Only slight body weight loss after each injection were observed and then rapidly recovery within 2 days.

To assess the therapeutic immune regulation, we further investigate the ratios of CD4^+^ and CD8^+^ T cells after 14 and 28 days after first treatment of Pembrolizumab mRNA-LNPs. The results of flow cytometry analysis showed the modestly increased the frequency of CD4^+^ and CD8^+^ T cells, whereas the ratios of CD8/Treg and CD8/CD4 cells were increased in groups received the treatment of all three mRNA-LNPs compared to the empty LNPs (Figure 6C).

## Discussion

Recently, RNA-based therapies have been widely applied in numerous clinical trials, including Infectious disease vaccines, protein replacement therapies, personalized tumor vaccines, and gene editing therapies 26, 27. Specifically, application of LNP-formulated mRNAs can not only effectively address the disadvantages of bi-specific antibodies, including short *in vivo* lifespan, but also target intracellular targets. Moreover, for treating the hepatopathy, the therapeutic window can also be improved by reducing the chance of systemic exposure through *in situ* synthesis and secretion by the liver. Delivery of mRNA prepared via IVT technology in the body can realize the expression of antibodies or proteins and overcome chemical manufacturing and control (CMC)-related challenges 28, 29, and CMC challenge 28. In addition, endogenous production of mRNA-translated antibodies makes low cost and simple manufacturing process compared to antibodies produced via host translational machinery 30. More importantly, the application of LNP-mRNAs can enable local expression of antibodies or proteins without the need of structure optimization process 12. Previously reported data demonstrated the successful application of IVT-mRNA for endogenous expression of antibodies and exhibited prominent advantages in protecting humanized mice from HIV-1 challenge and selectively reduced the volume of HER2-positive tumors 10, 30.

In present research, we tried to broaden the application of LNP-based delivery of mRNAs for endogenous expression of mAbs for treating solid tumor. Pembrolizumab is one of the most widely clinical used anti-tumor mAbs with fully understood action mechanisms 24. The key to mRNA therapy is itself and the delivery system. Since mRNA is easily degraded and has a stimulation of the innate immune system, mRNA is very susceptible to many aspects such as gene sequence modification, delivery system, and production process. In addition to negatively charged mRNA, LNP encapsulates four components: ionizable cationic phospholipids, neutral auxiliary phospholipids, cholesterol, and polyethylene glycol-modified phospholipids (PEGylated lipid). The effect of excipients in nanoparticles is similar to the effect of such excipients in liposomes: neutral auxiliary phospholipids are generally saturated phospholipids, which can increase the phase transition temperature of cationic liposomes, support the formation of lamellar lipid bilayers and stabilize their structural arrangement; cholesterol has strong membrane fusion and promotes mRNA intracellular intake and cytoplasmic entry; PEGylated phospholipids are located on the surface of nanoparticles, improve their hydrophilicity, avoid rapid clearance by the immune system, prevent particle aggregation, and increase stability. The most critical excipient is the ionizable cationic phospholipid, which is a decisive factor in mRNA delivery and transfection efficiency.

In current study, we firstly optimized the SP sequences and HC/LC ratio of mRNA for maximizing expression levels of intact Pembrolizumab antibody *in vitro* and *in vivo*. As a result, current optimization on the usage of SP and HC/LC ratio achieved significanly improvement on the expression level of Pembrolizumab *in vivo*. Not only that, a series of *in vitro* and *in vivo* experiments were also conducted to confirm whether the *in vivo* mRNA-translated Pembrolizumab maintain the functionality, and whether also can suppress the tumor growth and extend survival of MC38 tumor-bearing mice. As we all know, the potent anti-PD-1 antibody requires specific and strong binding affinity to the human PD-1 and the blocking potency of PD-1 to bind with PD-L1 and PD-L2. Therefore, whether the mRNA translated Pembrolizumab can retain the similar affinity to PD-1 and its ligands blocking property were assessed. As showed in Figures 4A-B, SPR measurement exhibited that the Pembrolizumab from two sources showed comparable binding affinities to human PD-1. Similar results of the binding and ligands blocking potencies were also confirmed by using the cell-based binding/blocking assay (Figures 4E-F). Comparable effects of Pembrolizumab from *in vivo* mRNA-translation and CHO cells expression on the activation of T cells activation were indicated by the release of IFN-γ and IL-2 in the MLR assay (Figure 4). Above data collectively indicated that the Pembrolizumab from both two sources harbor similar functional pharmacological specifications *in vitro*.

We further investigated whether the delivery of optimized Pembrolizumab mRNA is capable of expressing intact antibodies in rodent animals. The normal C57BL/6 mice received the single injection of Pembrolizumab mRNA-LNPs at the doses of 0.2, 0.6 and 2 mg/kg and all three doses were well tolerated without obvious side effects. As showed in Figure 2A, a clearly dose-dependent upregulation of the serum level of Pembrolizumab at 6h, 24h, 48h and 72h after administration. The normal C57BL/6 mice received the single injection of Pembrolizumab mRNA at the doses of 0.2, 0.6 and 2 mg/kg were observed with serum levels in a dose dependent manner, of which the 2m/kg was comparable with injection of the Pembrolizumab protein at 10 mg/kg. Significantly, we observed that the obviously longer Pembrolizumab persistence in the blood circulation of mouse after the single i.v. delivery of LNP-mRNAs compared with that of direct treatment of Pembrolizumab proteins. After single injection of 40 μg LNP-mRNAs or 200μg proteins, the serum level of endogenously translated Pembrolizumab was comparable with that of administrated antibodies at 24 h. Then, the Pembrolizumab levels in the ones received antibodies continuously declined to baseline within 21 days, while that of mice administrated with LNP-mRNAs was still rising until 72 h (C_max_~65 μg/mL), and then remained over 40 μg/mL for at least 3 weeks, which is regarded as over necessary therapeutic serum concentration for antitumor efficacies. It is worth mentioning that anti-drug antibody (ADA) responses were not observed, which may be that only single injection of mRNA-LNPs encoding humanized antibodies in mice was performed in current test, and not repeated injections. However, immunodeficient mice were used for further repeated injection tests, mainly because of the potential immunogenicity of humanized antibodies expressed from mRNA-LNPs under three repeated injections.

Current study firstly demonstrated the inhibitory effects of delivery of mRNA encoded Pembrolizumab on solid tumor growth. Interestingly, we did not observe any significant toxic effects during experimental period. Further validation of anticancer efficacy test demonstrated that the i.v. injection of 0.2, 0.6 and 2.0 mg/kg of Pembrolizumab mRNA-LNPs for 6 weeks can obviously inhibit the growth of colorectal carcinoma, and significantly improved the survival rate of tumor bearing mice (Figure 6). Not only that, the prolonged persistence of endogenously translated Pembrolizumab in serum holds potential to decrease the clinical dose, injection frequency as well as the treatment costs.

It's worth discussing that the prolonged mAbs duration mainly due to two main reasons: 1) mRNA can be continuously expressed in hepatocytes for at least 72 hours, which can achieve the phased continuous release of mRNA-encoded antibodies into the blood circulation; 2) In mice, mRNA-encoded antibodies are endogenous antibodies, which may be endogenous post-translational modifications that bring better stability. In short, serum levels of endogenously expressed antibodies tend to be sustained longer than the purified agents, which is mainly due to the differences in the delivery method, translated efficiency as well as endogenous modification.

In summary, current research demonstrated that the application of IVT-prepared mRNA formulated into LNPs can effectively express endogenous therapeutic antibodies, not only intact Pembrolizumab, in hepatocytes which provide an alternative to antibody treatment for treating cancer treatment. Current research also provided a clear research approach for the optimizing the translated and secretory efficiency of mRNA, which could also be applied for the development of other mRNA encoding mAb-based therapies.

## Author Contributions

**Conceptualization:** Shihai Xuan, Anquan Shang; **Data curation:** Lipei Wu, Weiwei Wang, Jiale Tian; **Methodology:** Lipei Wu, Weiwei Wang, Jiale Tian, Chunrun Qi, Zhengxin Cai; **Resources:** Shihai Xuan, Anquan Shang; **Software:** Zhengxin Cai, Wenhui Yan; **Supervision:** Shihai Xuan, Anquan Shang; **Validation:** Lipei Wu, Weiwei Wang, Jiale Tian, Chunrun Qi; **Writing-original draft:** Lipei Wu; **Writing - review & editing:** Weiwei Wang, Jiale Tian, Chunrun Qi, Zhengxin Cai, Wenhui Yan, Shihai Xuan, Anquan Shang.

## Abbreviations

IVT: *in vitro* transcription
LNP: lipid nanoparticle
hPD-1: human PD-1
ITS-G: Insulin-Transferrin-Selenium
Pen/Strep: penicillin-streptomycin
FBS: fetal bovine serum
TGI: tumor growth inhibition
IE1: cytomegalovirus immediate early 1
SP: signal peptide
hIgLC: human immunoglobulin kappa light chain
hIgHC: human IgG1 heavy chain
AUC: area under the curve
Cl: serum clearance
Vss: steady-state distributed volume
MLR: mixed lymphocyte reaction
